# *COLQ*-Congenital myasthenic syndrome in an Iranian cohort: the clinical and genetics spectrum

**DOI:** 10.1186/s13023-024-03116-x

**Published:** 2024-03-12

**Authors:** Omid Hesami, Mahtab Ramezani, Aida Ghasemi, Farzad Fatehi, Ali Asghar Okhovat, Bentolhoda Ziaadini, Ariana Kariminejad, Shahriar Nafissi

**Affiliations:** 1 Neuromuscular Research Center, Tehran University of Medical Sciences, Tehran, Iran; 2 Neurology Department, Shariati Hospital, Tehran University of Medical Sciences, Tehran, Iran; 3 Neurology Department, Imam Hossein Hospital, Shahid Beheshti University of Medical Sciences, Tehran, Iran; 4 Neurology Research Center, Kerman University of Medical Sciences, Kerman, Iran; 5 Kariminejad-Najmabadi Pathology and Genetics Center, Tehran, Iran

**Keywords:** Congenital myasthenic syndrome (CMS), *COLQ* gene, Acetylcholinesterase-associated collagen protein, Muscle weakness, Cholinesterase inhibitors

## Abstract

**Background:**

Congenital myasthenic syndrome (CMS) is a group of neuromuscular disorders caused by abnormal signal transmission at the motor endplate. Mutations in the collagen-like tail subunit gene (*COLQ*) of acetylcholinesterase are responsible for recessive forms of synaptic congenital myasthenic syndromes with end plate acetylcholinesterase deficiency. Clinical presentation includes ptosis, ophthalmoparesis, and progressive weakness with onset at birth or early infancy.

**Methods:**

We followed 26 patients with *COLQ*-CMS over a mean period of 9 years (ranging from 3 to 213 months) and reported their clinical features, electrophysiologic findings, genetic characteristics, and therapeutic management.

**Results:**

In our population, the onset of symptoms ranged from birth to 15 years. Delayed developmental motor milestones were detected in 13 patients ($\sim$ 52%), and the most common presenting signs were ptosis, ophthalmoparesis, and limb weakness. Sluggish pupils were seen in 8 ($\sim$ 30%) patients. All patients who underwent electrophysiologic study showed a significant decremental response (> 10%) following low-frequency repetitive nerve stimulation. Moreover, double compound muscle action potential was evident in 18 patients ($\sim$ 75%). We detected 14 variants (eight novel variants), including six missense, three frameshift, three nonsense, one synonymous and one copy number variation (CNV), in the *COLQ* gene. There was no benefit from esterase inhibitor treatment, while treatment with ephedrine and salbutamol was objectively efficient in all cases.

**Conclusion:**

Despite the rarity of the disease, our findings provide valuable information for understanding the clinical and electrophysiological features as well as the genetic characterization and response to the treatment of *COLQ*-CMS.

**Supplementary Information:**

The online version contains supplementary material available at 10.1186/s13023-024-03116-x.

## Introduction

Congenital myasthenic syndrome (CMS; OMIM #603,034) refers to a heterogeneous group of rare inherited disorders affecting neuromuscular transmission [1]. These syndromes are categorized into presynaptic, synaptic, and postsynaptic disorders based on the location of the neuromuscular junction defect [2, 3]. The clinical manifestations vary depending on the age at onset (AAO) and are mainly characterized by fatigable muscle weakness and respiratory failure. The diagnosis of CMS can be made through clinical and electrophysiologic findings, along with the absence of acetylcholine receptor or muscle-specific tyrosine kinase antibodies, lack of symptom improvement with immunosuppressive therapy, and often a positive family history. However, accurate genetic analysis is particularly important for identifying the molecular pathways that will lead to a better understanding of biological mechanisms and disease pathophysiology and consequently provide guidance on selecting the best medication [4, 5]. To date, approximately 35 cm-causing genes have been identified, most of which are inherited in an autosomal recessive manner [6]. The causative genes can be categorized into 14 distinct groups based on their pathomechanical characteristics [6].

Synaptic CMS is mostly caused by variants in the *COLQ* gene (collagen-like tail subunit of asymmetric acetylcholinesterase, OMIM #603,033), which encodes a collagen-like strand that associates into a triple helix to constitute a tail that anchors catalytic subunits of acetylcholinesterase (AchE) to the basal lamina [1, 6, 7]. The *COLQ*-CMS is predominantly present with early-onset progressive limb weakness, axial weakness, and respiratory insufficiency. The presence of a slow pupillary light response, repetitive compound muscle action potentials (CMAPs) in nerve conduction and repetitive nerve stimulation (RNS) studies, and the absence of improvement or even worsening of symptoms following AChE inhibitor treatment are considered hallmarks for diagnosis [6].

While the occurrence of *COLQ*-CMS is infrequent, the initial manifestations may be mistaken for myasthenia gravis. However, with timely and accurate diagnosis, as well as prompt treatment, there is a possibility of enhancing the functional ability and overall quality of life of the affected individuals.

Herein, we studied a series of Iranian *COLQ*-CMS patients and described their clinical and genetic findings, as well as their response to treatment. To the best of our knowledge, this is one of the largest numbers of CMS patients with *COLQ* gene variants that were compiled in a single study and followed for several years, allowing assessment of disease course and response to treatment.

## Methods

### Patient recruitment and clinical evaluation

This research was performed in accordance with the Declaration of Helsinki and with the approval of the ethics board of the Tehran University of Medical Sciences in Iran. The clinical details were collected from the patient upon obtaining written informed consent.

In this retrospective study, the genetically confirmed patients were followed over a mean period of 9 years (ranging from 3 to 213 months). Participants were recruited from three neuromuscular centers in Tehran. Patient 15 has been previously reported [8]. Detailed descriptions of clinical features, sex, AAO, age at diagnosis, ethnicity, duration between onset and diagnosis, and consanguinity were recorded. The patients were examined by manual muscle testing using the six-point Medical Research Council (MRC) scale (ranging from 0 to 5). Moreover, the MRC-sum scores of the neck extensor, neck flexor, shoulder abduction, elbow flexion, wrist extension, hip flexion, knee extension, and ankle dorsiflexion muscles on both sides (sum score = 0–70) were calculated. All patients, except two, underwent nerve conduction studies (NCVs) as well as low-frequency RNS on the abductor pollicis brevis, abductor digiti minimi, anconeus, trapezius, and facial muscles.

### Whole exome sequencing and data analysis

DNA from 21 family members was extracted from peripheral blood samples. Whole exome sequencing (WES) was performed for each proband using the SureSelect Human All Exon V6 (Agilent Technologies Inc., Santa Clara, CA, USA) enrichment kit. Preliminary filtering was performed to detect all homozygous variants based on the recessive pattern of inheritance. Thereafter, variants that did not affect amino acid chains or splicing sites and SNPs with a minimal allele frequency > 0.01 in the public databases were filtered out. The remaining variants were evaluated based on the American College of Medical Genetics (ACMG) criteria [9]. In parallel, copy number variation (CNV) detection based on WES data was performed. The GATK method used the Determine Germline Contig Ploidy module to determine autosomal and allosomal contig ploidy. Then, the GermlineCNVCaller algorithm was used to detect CNVs.

### Variant confirmation

The candidate single number variant (SNV) variants in the *COLQ* gene were amplified from the DNA of the probands by polymerase chain reaction (PCR). The PCR products were sequenced using the Sanger method. Sequences were analyzed by comparison with the reference sequence available at NCBI: NC_000003.11, NM_005677.4, and NP_005668.2 for the *COLQ* gene. After confirming the variants in the probands, cosegregation analysis was performed for the family members. Multiplex ligation-dependent probe amplification analysis (MLPA) was also performed to confirm the large deletion in patient P 14.

### Treatment

Ephedrine was given to 15 patients, while salbutamol was administered to 11 patients. We evaluated the patients before starting treatment and after 3 and 6 months using the Myasthenia Gravis Activities of Daily Living scale (MGADL), which is a validated eight-item questionnaire. Each item is scored from 0 (normal) to 3 (most severe), providing a total MGADL score ranging from 0 to 24. A higher score indicates a greater severity of symptoms.

## Results

**Fig. 1:**
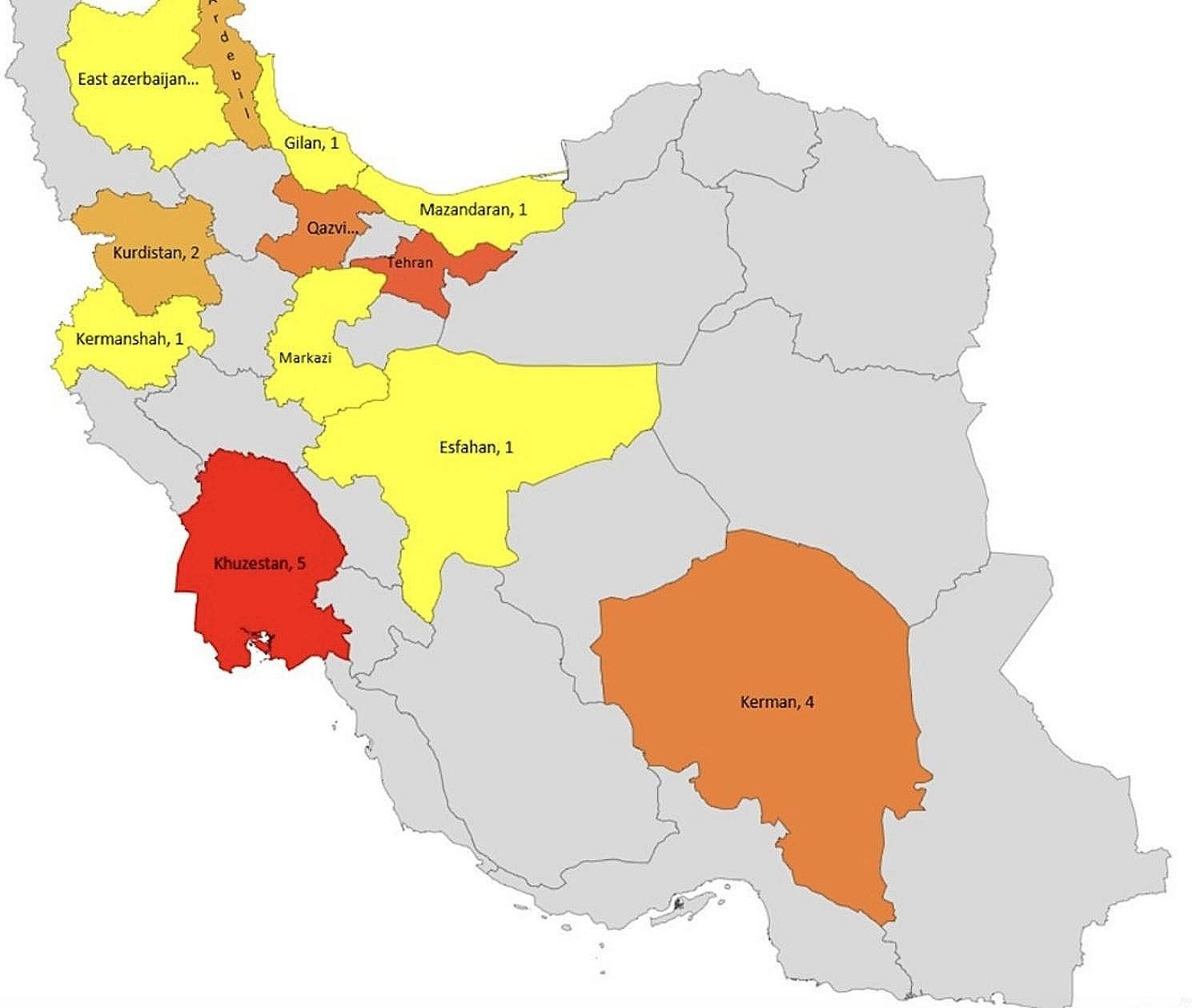
Geographical origin of 26 Iranian patients with COLQ-CMS

In this study, 26 patients from 21 unrelated Iranian families were recruited, including nine ($\sim$ 35%) women and 17 ($\sim$ 65%) men. All patients were from mainland Iran and were distributed across nine provinces (Fig. 1). Among the patients, 23 ($\sim$ 88%) were born to consanguineous parents, 16 ($\sim$ 64%) had a positive family history, and the median age of patients at the first visit was 9 years old, with a range of 8–39 years. patients 1.1 & 1.2, 5.1 & 5.2, 7.1 & 7.2, and 9.1 & 9.2 are siblings. The majority of patients ($\sim$ 57%) manifested symptoms at birth, and the median duration of diagnosis from symptom onset was 9 (3–36) years.

### Clinical characteristics

More than half of the patients had disease onset at birth. Eleven ($\sim$ 42%) patients manifested in childhood or adolescence, with a median age of 9. The latest disease AAO was 15 years old. The most common presenting signs at birth were ptosis, ophthalmoparesis, and flaccid limb weakness. Two patients (P5.1 and P7.2) reported fatigue as their initial symptom. We observed delayed developmental motor milestones (DDMM) in 14 patients ($\sim$ 53%). All patients with DDMM achieved walking ability in our study; however, the age at which they obtained this ability varied among the cases. Facial weakness was found in 20 patients, and bilateral ptosis and ophthalmoparesis were found in 17 and 15 patients, respectively. A slow pupillary response to light was detected in only 8 ($\sim$ 30%) patients, and a high-arched palate was noted in six ($\sim$ 23%) of our patients. All patients underwent pulmonary function tests during their visit to the neuromuscular clinic, and we repeated the test based on clinical findings. Three patients (P1.2, P10, and P11) experienced severe respiratory distress and clinical signs of hypoventilation that required Bilevel Positive Airway Pressure support (BiPAP). The median MRC-sum score was 56 (40–62), and 21 (80%) had axial muscle involvement. Diurnal variation was evident in 16 patients, and 7 of 26 patients ($\sim$ 26%) showed progression of symptoms, including limb weakness and ptosis. Families 1, 2, 16, and 17 each had a sibling with ptosis/muscle weakness and respiratory distress who died before the age of 2yrs. The demographics, clinical profiles, electrodiagnostic findings, and genetic characteristics of the patients are summarized in Table 1.

**Table 1:** Detailed clinical features of patients with COLQ gene variants

Clinical Findings																			
Patient ID	Sex	AAD (y)	AAO (y)	Consanguinity	Family History	DDMM	First Symptom	Follow-up Duration (m)	Progression	Diurnal Variation	Ptosis/ Ophtalmoparesis	Facial Weakness	Sluggish Pupils	High-arched Palate	PMH	MRC Sumscore	Repetitive CMAP	Decrement in LFRNS (%)	Needle EMG
P 1.1	F	36	At birth	+	+	+	Ptosis/ Limb Weakness	26	Steady	+	+/+	+	-	+	HTN	40	NA	NA	NA
P 1.2	F	31	At birth	+	+	+	Ptosis/ Limb Weakness	20	Steady	+	+/+	+	-	+	-	54	NA	NA	NA
P 2	F	39	9	+	-	-	Progressive Proximal Weakness	12	Progressive	-	-/-	-	-	-	HLP	58	-	33.20%	Myopathic
P 3	M	9	At birth	+	+	+	Floppy Baby	7	Steady	+	-/-	+	-	+	-	56	-	37.80%	Myopathic
P 4	M	19	At birth	+	+	+	Floppy Baby	67	Steady	-	+/+	+	-	-	-	59	+	39%	NA
P 5.1	M	20	9	+	+	-	Fatiguability/ Limb Weakness	92	Progressive	+	-/-	-	+	-	-	61	+	31.60%	Myopathic
P 5.2	M	11	8	+	+	-	Fatiguability	12	Progressive	-	+/-	+	+	-	-	68	+	11%	Myopathic
P 6	F	32	3	+	-	-	Ptosis/ Limb Weakness	48	Steady	+	+/+	+	-	+	-	61	+	43.90%	NA
P 7.1	F	9	At birth	+	+	+	Ptosis	6	Steady	-	+/+	+	+	-	-	51	+	43.50%	Myopathic
P 7.2	M	18	At birth	+	+	+	Ptosis/ Fatiguability	6	Steady	-	+/+	+	+	-	-	56	+	14%	Myopathic
P 8	F	13	7	+	-	-	Limb Weakness	19	Steady	-	-/-	-	-	-	Febrile Convulsion	58	+	35.90%	NA
P 9.1	M	15	9	+	+	-	Limb Weakness	18	Progressive	+	-/-	+	+	-	Minor Thalassemia	56	+	21.70%	NA
P 9.2	F	9	9	+	+	-	Limb Weakness	4	Steady	-	-/-	-	-	-	Minor Thalassemia	62	+	11.20%	NA
P 10	M	23	At birth	+	-	+	Floppy Baby	18	Progressive	-	+/-	-	+	-	-	53	+	40.70%	NA
P 11	M	22	At birth	+	+	+	Floppy Baby/ Ptosis	51	Steady	+	+/+	+	-	-	-	49	-	22.80%	NA
P 12	M	16	9	-	-	-	Walking Difficulty	54	Steady	+	-/-	+	-	-	-	60	+	42.20%	Myopathic
P 13	M	21	At birth	-	+	+	Floppy Baby/ Ptosis	52	Steady	+	+/+	+	-	-	-	56	+	71.40%	NA
P 14	F	22	At birth	-	+	+	Ptosis	153	Steady	+	+/+	+	-	-	Asthma/ Club foot	49	-	65.90%	NA
P 15	M	36	At birth	+	-	-	Ptosis/ Limb Weakness	177	Progressive	+	+/+	+	+	+	-	54	+	45.30%	NA
P 16	M	16	At birth	+	+	+	Ptosis/ Limb Weakness	13	Steady	-	+/+	+	-	+	-	56	+	65.60%	Normal
P17	M	27	15	+	-	-	Walking Difficulty	213	Steady	+	-/-	+	-	-	-	56	+	35.20%	Myopathic
P 18	M	27	At birth	+	+	+	Limb Weakness	134	Steady	+	+/+	+	-	-	-	55	-	34.70%	Myopathic
P 19	M	9	At birth	+	+	+	Limb Weakness	17	Steady	+	+/+	+	-	-	-	58	-	21.60%	Myopathic
P 20	M	17	8	+	-	-	Limb Weakness	28	Steady	+	-/-	+	-	-	-	59	+	24%	Myopathic
P 21	F	27	3	+	-	-	Ptosis/ Limb Weakness	35	Steady	-	+/+	-	-	-	-	54	+	35.60%	Myopathic
P 22	M	8	At birth	+	-	+	Ptosis	3	Progressive	+	+/+	+	+	-	-	60	+	42.40%	Myopathic

M: Male, F: Female, AAD: age at diagnosis, AAO: age at onse, DDMM: Delayed Developmental Motor Milestone, NA: Not Available, PMH: Past Medical History, HTN: Hypertension, HLP: Hyperlipidemia, MRC: Medical Research Council, LFRNS: Low Frequency Repetitive Nerve Stimulation

### Electrodiagnostic findings

As illustrated in Table 1, NCS and RNS studies were carried out on 24 patients. A significant decremental response (> 10%) following the low-frequency RNS study was noted in all 24 (100%) patients in at least two tested muscles. The mean decremental response of the abductor digiti minimi muscle was $\sim$ 40% (range: 11-71.40%). Repetitive CMAP in response to single nerve stimulus and RNS study was evident in 18 patients ($\sim$ 75%). Needle electromyography was executed in 14 patients, with all but one revealing early recruitment of short-duration motor unit potentials without any spontaneous activity.

### Genetic findings

A total of 14 variants of the *COLQ* gene in 26 patients were identified, including eight novel variants, which are summarized in Table 2. Among these variants, six were missense, three were frameshift, three were nonsense, one was a CNV, and one was synonymous, the latter of which could potentially impact splicing. Based on the ACMG criteria, six variants were classified as “pathogenic”, five as “likely pathogenic” and three as “variance of uncertain significance” (VUS). The most common variants were c.1277 C > T:(p. Thr426Ile) and c.1082del:(p. Pro361LeufsTer65), each found in five patients. All the variants were submitted to the ClinVar database.

**Table 2:** Genetic Findings of COLQ_CMS patients (NM_005677.4)

Patient ID	Variant; cDNA level	Variant; Protein level	Zygousity	ACMG classification
P 1.1, P1.2	c.1082del	p.Pro361LeufsTer65	Hom	Pathogenic
P 2	†c.1132G > C	p.Gly378Arg	Hom	VUS
P 3	c.1257del	p.Ser420LeufsTer6	Hom	Likely Pathogenic
P 4	c.211 C > T	p.Arg71Ter	Hom	Likely Pathogenic
P 5.1, P5.2	†c.1277 C > T	p.Thr426Ile	Hom	Pathogenic
P 6	†c.1196G > A	p.Arg399His	Hom	Pathogenic
P 7.1, P7.2	†c.815G > A	p.Gly272Glu	Hom	VUS
P 8	c.1026 C > G	p.Asp342Glu	Hom	Likely Pathogenic
P 9.1, P9,2	†c.1277 C > T	p.Thr426Ile	Hom	Pathogenic
P 10	†c.1076T > G	p.Leu359Arg	Hom	VUS
P 11	c.1082del	p.Pro361LeufsTer65	Hom	Pathogenic
P 12	†c.1257del	p.Ser420LeufsTer6	Hom	Likely Pathogenic
P 13	c.1082del	p.Pro361LeufsTer65	Hom	Pathogenic
P 14	†c.188_321del	del ex 2–3	Hom	Likely Pathogenic
P 15	c.679 C > T	p.Arg227Ter	Hom	Pathogenic
P 16	c.943 C > T	p.Arg315Ter	Hom	Pathogenic
P17	c.1281 C > T	p.Cys427=	Hom	Pathogenic
P 18	c.943 C > T	p.Arg315Ter	Hom	Pathogenic
P 19	†c.827_843del	p.Met276LysfsTer20	Hom	likely Pathogenic
P 20	†c.1277 C > T	p.Thr426Ile	Hom	Pathogenic
P 21	†c.1196G > A	p.Arg399His	Hom	Pathogenic
P 22	c.1082del	p.Pro361LeufsTer65	Hom	Pathogenic

VUS: Variant of Undetermined Significance, Hom: Homozygous, † Novel Variant

### Treatment

We would like to clarify that patients in this study were treated by their physicians according to the standard of care. As our study is retrospective, the assignment to a treatment group was not pre-determined, but rather based on routine medical practice.

Regarding the pyridostigmine effect, 11 patients experienced a clear worsening of symptoms, while eight patients had no change or beneficial effects. Ephedrine (30–90 mg/Kg/day divided into two to three doses, not exceeding 150 mg per day) was administered to 15 patients, and 13 of them (86.6%) reported an improvement in clinical symptoms. In the responder group, ten patients showed at least a 2-point improvement in MG-ADL scores. Salbutamol (2–12 mg/day divided into two to four doses) was given to 18 patients. Due to the shortage of ephedrine in our country for a long period of time, ten patients were switched from ephedrine to salbutamol treatment. After six months of drug administration, all patients exhibited clinical improvement, with 15 indicating a reduction of 2 points or more in the MG-ADL scoring system. The response to treatment is shown in Table 3.

**Table 3:** Response to treatment in COLQ-CMS patients

Tx	P1.1	P1.2	P2	P3	P4	P5.1	P5.2	P6	P7.1	P7.2	P8	P9.1	P9.2	P10	P11	P12	P13	P14	P15	P16	P17	P18	P19	P20	P21	P22
Pyridostigmine	NT	NT	W	NT	NT	NC	NC	NC	NT	NT	W	NC	NC	W	W	W	NT	W	W	W	W	W	W	NC	NC	NT
Ephedrine	+	+		+	+	+			+	+		+			+	+	+		+	+		+	+			
ΔMGADL for Ephedrine Tx	1	2		1	2	1			1	1		3			2	3	2		4	2		5	2			
Salbutamol Response			+		+	+			+	+	+	+		+	+		+	+	+		+	+	+	+	+	+
ΔMGADL For Salbutamol Tx			3		1	1			2	1	4	2		2	2		2	3	2		3	3	2	2	2	2

MGADL: Myasthenia Gravis Activities of Daily Living scale; Tx: treatment; NC: no change; W: worsening; NT: not tried

## Discussion

The *COLQ* gene plays a crucial role in anchoring and accumulating AChE at neuromuscular junctions. Mutations in the *COLQ* gene can lead to AChE deficiency and an increased half-life of acetylcholine (ACh) in the synaptic cleft. Consequently, AChs can bind multiple times to acetylcholine receptor before leaving the synaptic space, leading to prolonged end-plate potentials and the generation of a second muscle action potential. This creates a repetitive CMAP, which can be a characteristic feature [8, 10]. Since *COLQ*-CMS is usually present at birth or during infancy, DDMM was a usual event in these patients. This finding has been confirmed in two previous large cohorts of *COLQ*-CMS conducted in 2008 and 2012 [8, 11]. Our findings were also compatible with a high prevalence of delayed motor milestones; moreover, five individuals ($\sim$ 19%) exhibited generalized hypotonia at birth. However, prior studies have suggested that arthrogryposis, or floppy baby at birth, is not a common occurrence [8].

Wargon et al., in a ten-year follow-up study of 15 *COLQ-*CMS cases, demonstrated fluctuation in muscle weakness and was sometimes associated with respiratory issues in nine (60%) patients. The authors identified that the patients experienced short- and long-term worsening of symptoms following esterase inhibitors, effort, puberty, or pregnancy [8]. Moreover, in a study conducted by Mihaylova et al., they followed 22 *COLQ*-CMS patients and showed diurnal fluctuation in eight (36%) cases [7]. According to our data, 61% of cases exhibited fatigability and daily fluctuations in their symptoms.

We observed that ocular muscle involvement was frequently seen at the onset of symptoms. Bilateral ptosis was present in 17 ($\sim$ 65%) patients, and extraocular movements were limited in 15 ($\sim$ 57%) cases, as shown in Table 1. A recent review carried out in 2023 on *COLQ*-CMS patients revealed that 90 out of 120 cases suffered from ptosis, while $\sim$ 50% of patients showed ophthalmoparesis, with only five patients having complete ophthalmoplegia [12]. Although sluggish pupils are one of the clinical clues for the diagnosis [13], they were observed only in $\sim$ 30% of our patients. Facial weakness was a common feature in our study and was seen in 20 patients. Eshaghian et al., in their review, also reported sluggish pupils and facial palsy in 15.6% and 84% of cases, respectively [12].

Most patients in the current study experienced generalized weakness. Patient 5.2 was an 11-year-old boy who complained of fatigability and only had proximal lower limb weakness in muscle force examination. In general, proximal muscles were more frequently affected than distal muscles. This is consistent with previous research that identified proximal weakness as a common feature [12].

Twenty-four patients underwent an electrophysiological study, all of whom showed evidence of impaired neuromuscular transmission demonstrated by a decremental response to RNS. Similar to our observation, the two previous cohorts indicated significant decremental responses in approximately 90% of patients [7, 8]. In addition, we observed that $\sim$ 75% of our cases displayed double CMAPs, while in the Wargon et al. study, repetitive CMAPs were detected in all patients.

**Fig. 2:**
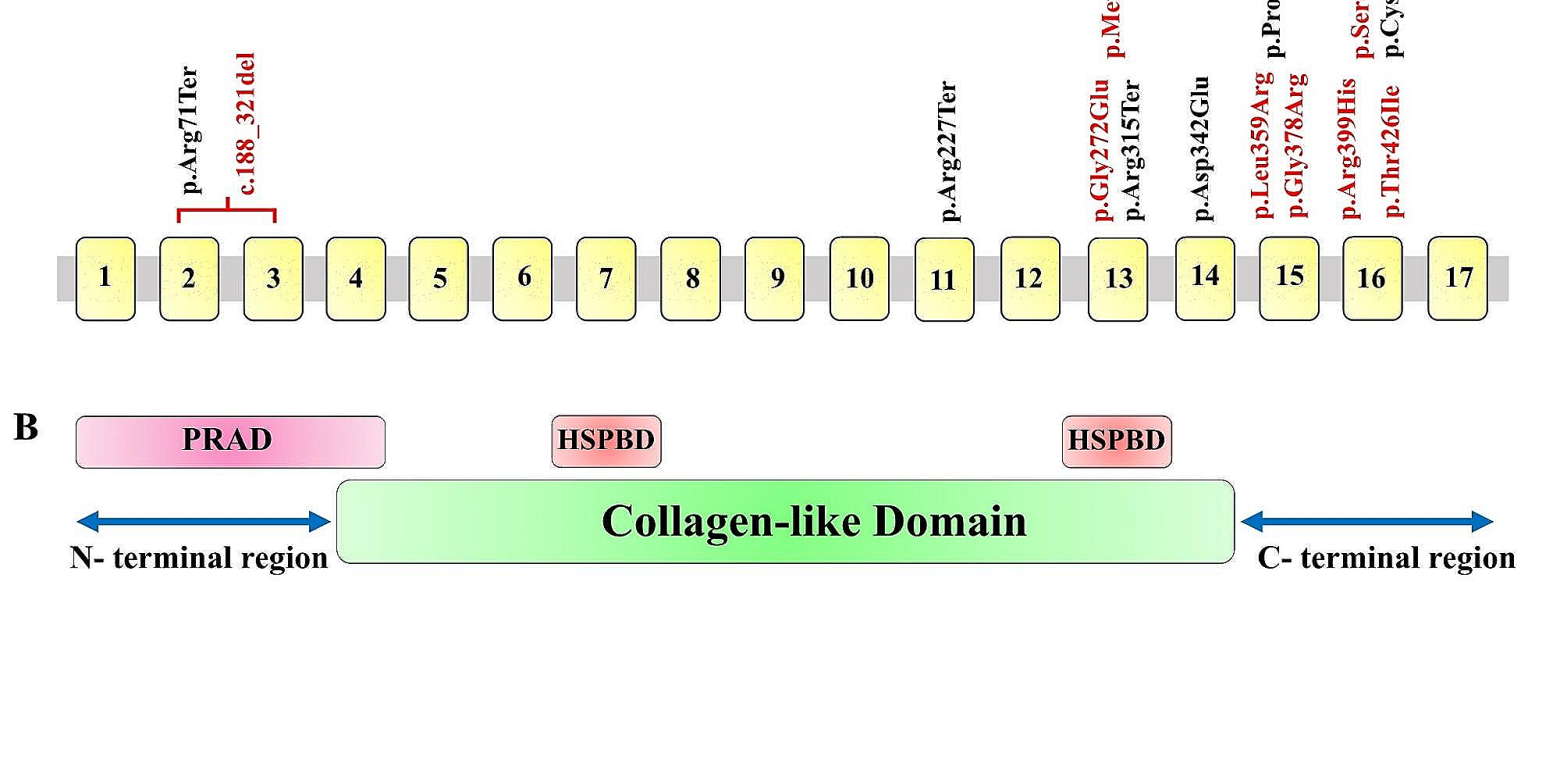
**(A)** Mutations in the *COLQ* gene identified in our patients. The yellow boxes represent the exons but do not reflect the exon length. The novel mutations are marked in red. **(B)** Three COLQ domains [1]: conserved domains of COLQ include an *N*-terminal proline-rich attachment domain (PRAD) [2], a central collagen domain that contains two heparan sulfate proteoglycan binding (HSPBD) domains, and [3] a C-terminal region

The *COLQ* gene, located on chromosome 3p25, spans approximately 50 kb and consists of 17 constitutive exons (Fig. 2A). Mutations in the *COLQ* gene are found in three parts of the COLQ protein: the proline-rich attachment domain (PRAD) located in the N-terminal region spanning from exon 1 to exon 4, the heparan sulfate proteoglycan-binding domain (HSPBD) located in the collagen-like domain spanning from exon 4 to exon 14, and the C-terminal region, which is encoded by genomic exons 15 to 17 (Fig. 2B) [3, 14]. To date, a total of 86 variants of the *COLQ* gene have been described in the HGMD database (professional 2023.1). According to Eshraghian et al.‘s review, the c.1289 A > C variant is the most commonly found variant in European populations, while c.444G > A is prevalent in Asian individuals [12]. However, in our cohort, the most repeated variants were c.1277 C > T and c.1082del. The review also demonstrated that missense variants are responsible for approximately 35% of alleles and cause a less severe phenotype in patients who carry them. On the other hand, splice variants can cause the most severe phenotype and are responsible for approximately 15% of patients. Moreover, nonsense, indel, and CNVs account for about 26%, 21%, and 2% of patients, respectively. In our cohort, $\sim$ 46% of patients carried missense variants. Frameshift variants are observed in $\sim$ 30%, and nonsense variants account for $\sim$ 15% of cases. Consistent with previous research, all patients who had frameshift and nonsense mutations, except for one, experienced severe symptoms from birth and had a higher MRC sum score. Conversely, one patient with a missense mutation showed symptoms at birth. Moreover, almost all individuals with a missense mutation, except one, exhibited repetitive CMAP in NCS. However, of seven patients with frameshift mutations, only 3 had double CMAP. One patient showed a synonymous variant of c.1281 C > T in the *COLQ* gene, who was a 27-year-old male patient who presented with walking difficulty at the age of 15 with an MRC sum score of 56. Wargon et al. reported the first patient with the c.1281 C > T variant in 2012. The authors proposed that the mutation causes a synonymous substitution, p.Cys427=, leading to abnormal splicing and the removal of 19 nucleotides from exon 16 [8].

Identifying the accurate causative gene in CMS is crucial to avoiding medication that worsens symptoms. Anticholinesterase medications such as pyridostigmine have been found to either worsen symptoms or offer no benefit in patients with *COLQ*-CMS. On the other hand, it has been reported that 3,4-diaminopyridine, ephedrine, and salbutamol can help treat *COLQ*-CMS [2, 5]. According to our study, $\sim$ 26% of patients experienced no positive effects from cholinesterase inhibitor treatment, while $\sim$ 42% experienced a worsening of their condition. Fifteen of our patients showed a significant improvement with ephedrine based on their MGADL score. Furthermore, the salbutamol trial resulted in a noteworthy change in the MGADL score. To summarize, as almost 75% of our cases presented with repetitive CMAPs in either the NCS or RNS study, the presence of double CMAP should raise concerns about COLQ-CMS. Therefore, clinicians should be cautious when prescribing pyridostigmine.

Our study has some limitations. There are several reasons for the delay between the onset of symptoms and diagnosis. Firstly, most patients were initially visited by a general neurologist before being referred to a neuromuscular clinic. Many of them were initially diagnosed with myasthenia gravis and were treated with pyridostigmine. Secondly, some of our patients refused to undergo genetic testing early in the course of the disease. Additionally, as indicated by Ciuffreda KJ et al., slow pupillary responses are characterized by initial constriction lasting more than one second and dilation lasting more than 5 s [15], and this definition is employed to identify sluggish pupils. However, there is a lack of uniform definitions of sluggish pupils across studies.

## Conclusion

In conclusion, we describe and discuss the clinical and genetic features as well as the response to treatment of Iranian *COLQ*-CMS patients. Our findings contribute to the growing number of mutations discovered in the *COLQ* gene. This highlights the significance of identifying genotypes to determine the appropriate treatment and provide proper genetic counseling to the family of this rare form of CMS. Additionally, the findings of this study indicate that in communities where consanguineous marriages are prevalent, such as Iran, the frequency of mutations in this gene may be higher than expected.

### Electronic supplementary material

Below is the link to the electronic supplementary material.

Supplementary Material 1

Supplementary Material 2

## Data Availability

The data that support the findings of this study are available from the authors but restrictions apply to the availability of these data, which were used under license from the Deputy of Research and Technology of Tehran University of Medical Sciences (TUMS) for the current study, and so are not publicly available. Data are, however, available from the authors upon reasonable request and with permission from the Deputy of Research and Technology of TUMS.

## Abbreviations

AAO: Age at onset
AchE: Acetylcholinesterase
ACMG: American College of Medical Genetics
AR: Autosomal recessive
BiPAP: Bilevel Positive Airway Pressure
CMAP: Compound muscle action potentials
CMS: Congenital myasthenic syndromes
CNV: Copy number variation
*COLQ*: Collagen-like tail subunit gene
DDMM: Delayed developmental motor milestones
HSPBD: Heparan sulfate proteoglycan-binding domain
MGADL: Myasthenia Gravis Activities of Daily Living scale
MLPA: Multiplex ligation-dependent probe amplification analysis
MRC: Medical Research Council
NCV: Nerve conduction studies
PCR: Polymerase chain reaction
PRAD: Proline-rich attachment domain
RNS: Repetitive nerve stimulations
SNV: Single number variant
VUS: Variance of uncertain significance
WES: Whole exome sequencing
