# Supplementary material for: COLQ-Congenital myasthenic syndrome in an Iranian cohort: the clinical and genetics spectrum
Source: Orphanet J Rare Dis. 2024 Mar 12;19:113. doi: 10.1186/s13023-024-03116-x (PMC10935773; doi:10.1186/s13023-024-03116-x)
Supplement: Supplementary file 1 — Supplementary Material 1 [file 13023_2024_3116_MOESM1_ESM.docx]

**Acknowledgements**

We acknowledge the Tehran University of Medical Sciences for funding the research and thank the patients and their family members for participating in the study.

**Conflicts of Interest**

The authors declare that the research was conducted in the absence of any commercial or financial relationships that could be construed as a potential conflict of interest.

**Informed consent**: Informed consent was obtained from all participants.

**Authors' contributions:** Sh. N, F. F, AA. O designed the manuscript, provided the outlines for the study's presentation, supervised the study process, and edited the final manuscript. A. GH & A. K assisted in the manuscript's genetic section and writing the pertinent section. M. R & O. H wrote the manuscript. All authors have reviewed the manuscript's data analysis process and writing and approved the final article.

**Data availability statement:** The data are available on request from the corresponding author.

**Funding:** No funding was received.
