# Supplementary material for: COLQ-Congenital myasthenic syndrome in an Iranian cohort: the clinical and genetics spectrum
Source: Orphanet J Rare Dis. 2024 Mar 12;19:113. doi: 10.1186/s13023-024-03116-x (PMC10935773; doi:10.1186/s13023-024-03116-x)
Supplement: Supplementary file 2 — Supplementary Material 2 [file 13023_2024_3116_MOESM2_ESM.docx]

| Supplementary Table. Bioinformatics based predictions by various softwares (In Silico Analysis) on functional effects of COLQ variations observed in the patients. | | | | |
| --- | --- | --- | --- | --- |
| Software | | | | |
| Patient | **Variation** | **Polyphen2_HDIV** | **Mutation Taster** | **CADD** |
| P1.1, P1.2, P11, P13, P22 | p.Pro361Leu | PrD (0.967) | DC (1) | 4.52 |
| P2 | p.Gly344Arg | PrD (0.994) | DC (0.999) | 3.38 |
| P3, P12 | p.Ser420Leu | PoD (0.453) | DC (0.999) | 1.42 |
| P4 | p.Arg71Ter | B (0.103) | DC (1) | 0.20 |
| P5.1, P5.2, P9.1, P9.2, P20 | p.Thr426Ile | PoD (0.773) | DC (0.999) | 1.87 |
| P6, P21 | p.Arg399His | PrD (0.986) | DC (0.908) | - |
| P7.1, P7.2 | p.Gly272Glu | PrD (1.000) | DC (0.999) | 0.85 |
| P8 | p.Asp342Glu | PrD (0.995) | DC (0.999) | 3.00 |
| P10 | p.Leu349Arg | PrD (0.995) | DC (0.999) | 4.38 |
| P14 | p.Pro63Arg | PrD (1.000) | - | 3.52 |
| P15 | p.Arg227* | - | DC (1) | 5.49 |
| P16, P18 | p.Arg315* | - | DC (0.999) | 1.00 |
| P17 | p.Cys427Cys | - | DC (1) | 0.56 |
| P19 | p.Met242Lys | - | DC (1) | 2.14 |

B: benign, D: damaging, PrD: probably damaging, PoD: Possibly Damaging, DC: disease causing, Del: deleterious.
